# Emotional Rational Education Training Associated with Mindfulness for Managing Anxiety within Adolescents Affected by High-Functioning Autism: A Descriptive Study

**DOI:** 10.3390/bs11110156

**Published:** 2021-11-11

**Authors:** Alessandro Frolli, Maria Carla Ricci, Francesca Di Carmine, Agnese Orefice, Emilio Saviano, Marco Carotenuto

**Affiliations:** 1 Disability Research Centre, University of International Studies in Rome, 00147 Rome, Italy; m.ricci@unint.eu (M.C.R.); f.dicarmine@unint.eu (F.D.C.); 2 FINDS-Italian Neuroscience and Developmental Disorders Foundation, 81040 Caserta, Italy; agneseorefice@hotmail.it (A.O.); emilio_saviano@live.com (E.S.); 3 Clinic of Child and Adolescent Neuropsychiatry, Department of Mental Health, Physical and Preventive Medicine, Università degli Studi della Campania “Luigi Vanvitelli”, 80100 Naples, Italy; marco.carotenuto@unicampania.it

**Keywords:** mindfulness and rational emotional education, autism spectrum disorder, internalizing disorders, social skills, education training, psychotherapy

## Abstract

Background: Autism spectrum disorder (ASD) is a chronic and persistent pervasive developmental disorder (PDD) whose characteristic deficit is represented by social difficulties, semantic–pragmatic alterations and a limited, unusual and repetitive pattern of interests and behaviors. Specifically, individuals with high-functioning autism (HFA) frequently exhibit associated internalizing symptoms that are not part of the diagnostic criteria but which, nonetheless, tend to impair daily functioning. In this study, we investigated how some forms of treatment could be useful in subjects with HFA who display internalizing symptoms. Theoretical background relates to standard cognitive therapy (SCT) and rational education training with mindfulness (M-ERE). Methods: In this study, we investigated how some forms of treatment could be useful in subjects with HFA and internalizing symptoms, focusing on standard cognitive therapy (SCT) and mindfulness associated with emotional rational education training (M-ERE). We selected two groups of HFA patients with significant internalizing symptoms and performed two different forms of treatment for six months: SCT and M-ERE. The aim of the study was to verify the effectiveness of an M-ERE protocol with respect to anxious and depressive symptoms in subjects with HFA. Furthermore, we wanted to compare the results obtained with this combined treatment with those obtained in HFA subjects treated with SCT. Results: Our analyses showed an improvement in the internalizing symptoms (especially those related to the anxiety dimension) of the group that followed a treatment based on mindfulness and rational emotional education for 6 months compared to the group that had instead performed a 6-month treatment based on the SCT. Conclusions: Our hypotheses were supported by the results, which highlighted the efficacy of mindfulness-based interventions in the treatment of internalizing symptoms in adolescents with HFA, and specifically showed that an M-ERE intervention appears more effective in managing anxiety compared to treatment with SCT and appears to be equally effective in the management of depressive symptoms. Not only was the M-ERE treatment effective for the management of anxious and depressive symptoms in subjects with HFA, but the efficacy for the management of anxious symptoms was greater than the SCT treatment.

## 1. Introduction

Asperger’s syndrome (AS) is a chronic and persistent pervasive developmental disorder (PDD) [1] whose characteristic deficit is represented by social difficulties, semantic–pragmatic alterations and an unusual and repetitive pattern of interests and behaviors. This syndrome differs from other pervasive developmental disorders due to the absence of clinically significant delays in cognitive development, language, adaptive behavior and autonomy. Asperger’s syndrome, first mentioned in DSM-IV [1], is no longer present in DSM-5 [2,3]; currently, individual situations previously classified as Asperger’s syndrome continue to exist clinically but fall within the level 1 autism spectrum disorders (ASD) [2]. In the present paper, we use the tag HFA.

In adolescence, social difficulties persist in these subjects; in particular, empathy, the modulation of social interactions, mentalization and more specifically the theory of mind (ToM) appear to be deficient. Individuals with HFA frequently show internalizing symptoms that are not part of the diagnostic criteria but which, nonetheless, tend to impair their daily functioning [4,5,6]. There are several hypotheses on the origins of the symptoms of anxiety and depression in ASD: according to some, they are associated with temperament or traits, such as behavioral inhibition towards family members [7,8], psychosocial distress or poor coping skills [8]. In fact, since reactions such as “sensory hyper-reactivity and excessive fear in response to harmless objects” are included in DSM-5 as associated characteristics of autistic disorder, these sensory alterations can enroll in an anxious temperament and play an important role in determining high levels of anxiety [5,9,10]. Social difficulties and lack of close relationships can also make individuals with HFA vulnerable to secondary affective symptoms similar to those that may arise in other individuals socially compromised (e.g., those with ADHD and conduct disorder) [11,12].

Regarding psychotherapeutic treatments for mood and anxiety disorders in individuals with HFA, few studies have been published in this regard. Most of the studies have been designed to address the associated symptoms of autism, difficulties and social skills, mentalization difficulties and emotional understanding [13]. However, such studies are not focused on secondary internalizing disorders (anxiety symptoms, depression, etc.) [14,15,16,17]. To the best of our knowledge, two randomized controlled trials (RCTs) treat anxiety in individuals with HFA and show some efficacy for children with HFA and anxiety compared to a control group [18,19]. Although the case studies suggest that cognitive-behavioral therapy (CBT) may also be effective within individuals with HFA and secondary internalizing symptoms [20,21], only few studies support this supposition. Furthermore, the symptoms and characteristics often associated with HFA would likely complicate the implementation of CBT procedures; for example, HFA individuals often suffer from a reduced ability to recognize thoughts and feelings both in themselves and in others (ToM) [22]. This would likely seem to hinder the introspection often required by CBT. Furthermore, deficits in language and social skills could prevent the formation of therapeutic relationships and the communication of complex or abstract concepts. These deficits on the whole would appear to hinder the effectiveness of CBT. More recently, mindfulness-based treatments have entered the spectrum of evidence-based treatments for anxious and depressive symptoms [23,24,25,26]. Mindfulness protocols revolve around two fundamental concepts: awareness of one’s actions and concentration on the present moment [24].

In this study, as an innovative element compared to previous studies, we investigated how some forms of treatment could be useful in subjects with HFA and internalizing symptoms and, for 6 months, we performed in the two groups of patients: group 1 was administered standard cognitive therapy (SCT) [27] while group 2 was administered a mindfulness intervention associated with emotional rational education training (M-ERE) [23,24,28,29,30,31]. In particular, we selected two groups of HFA patients with significant internalizing symptoms, and we performed the two different forms of treatment on the two groups of patients for six months: SCT and M-ERE. At the end of the treatment, we investigated any improvements or worsening of the internalizing aspects of the patients in order to verify the effectiveness of the interventions and make a comparison between them. The aim of the study was to verify the effectiveness of an M-ERE protocol with respect to anxious and depressive symptoms in subjects with HFA. Furthermore, we wanted to compare the results obtained with this combined treatment with those obtained in HFA subjects treated with SCT.

## 2. Materials and Methods

### 2.1. Participants

In this study, we considered a sample of 54 children who had been diagnosed with HFA (or also ASD Level 1), aged between 12 and 14 years who were patients at FINDS. The inclusion criteria were the following: (a) absence of other neurological, genetic or sensorineural pathologies; (b) cognitive functioning within the norm (=90)/above the norm (≥95), assessed through the administration of the Wechsler Intelligence Scale for Children-IV (WISC-IV) [32]; (c) absence of current or previously administered pharmacological treatments; (d) positive on the internalizing scale of the Youth Self-Report 11–18 (YSR) [33], of the Children’s Depression Inventory 2 (CDI 2) [34] and of the Screen for Child Anxiety Related Emotional Disorders (SCARED) [35], indicative of anxious and depressive symptoms. We divided the sample into two groups based on the type of training performed. Group 1 (Gr1) was composed of 27 subjects (6 females and 21 males; M = 13.50, SD = 0.70), while group 2 (Gr2) was composed of 27 subjects (7 females and 20 males; M = 13.30 SD = 0.55) (see Table 1). The data was collected by licensed psychologists at the Disability Research Center of the University of International Studies in Rome in collaboration with FINDS Child Neuropsychiatry Outpatient Clinic and the University of Studies of Campania “Luigi Vanvitelli”.

No sociocultural differences were found within the sample, as demonstrated by the results revealed through the implementation of the scale for the assessment of the socio-economic level (SES) [36] which was administered to both parents of the children. Specifically, the Gr1 reported a score of 7.3 (SD = 0.6), the Gr2 reported a score of 7.5 (SD = 0.3).

### 2.2. Instruments

The protocol used for the diagnostic evaluation consists of the following tests: Autism Diagnostic Observation Schedule–Module 3 (ADOS-2) [37], Krug Asperger’s Disorder Index (KADI) [38] and Diagnostic Interview for Evaluation of Psychopathological Disorders (K-SADS-PL DSM-5) [39]. The protocol used for the assessment of internalizing symptoms (anxious and depressive) consists of the following tests: Youth Self-Report 11–18 (YSR) [33], Children’s Depression Inventory 2 (CDI 2) [34] and Screen for Child Anxiety Related Emotional Disorder (SCARED) [35]. Furthermore, we used the scale for the assessment of the socio-economic level (SES) [36] to evaluate the socio-economic level.

***ADOS-2 (Module 3):*** It consists of standard activities that allow the examiner to observe the occurrence or not of behaviors that have been identified as important for the diagnosis of autism and other pervasive developmental disorders across developmental levels. Module 3 is designed for children who are verbally fluent. It consists of 14 activity sessions, in which, in addition to the imaginative and interactive game, the conversation and interview about emotions and friendships are provided.

***KADI:*** It consists of a screening tool built and structured to identify a possible Asperger’s disorder (or to exclude it), and to distinguish it from other forms of high-functioning autism, suitable for subjects aged between 6 and 21 years. The KADI is composed of 32 items that are administered individually to a person who lives in close contact with the subject (parents, teachers, educators, etc.) and who tries to identify those disorders that are relatively less serious than autism but characterized from an evident social disability.

***K-SADS-PL DSM-5:*** The K-SADS-PL consists in a diagnostic interview and detects the presence of psychopathological disorders in children and adolescents, referred to the criteria of DSM-5. It consists of the following parts: an unstructured introductory interview, a screening diagnostic interview, a checklist for administration of diagnostic supplements, five diagnostic supplements (mood disorders, psychotic disorders, anxiety disorders, attention deficit and disruptive behavior disorders, substance abuse disorders) for each of which the criteria required by DSM-5 are provided, one comprehensive checklist of the patient’s medical history and a scale for the overall assessment of the child’s current functioning. The K-SADS-PL allows to detect the presence of psychopathological frameworks and to code the symptoms of the subject.

***YSR:*** The YRS questionnaire is structured around eight syndromic scales: anxiety/depression, withdrawal/depression, somatic complaints, social disorders, thought disorders, attention disorders, rule-breaking behavior, aggressive behavior, which are grouped into two other general dimensions: internalizing and externalizing disorders. It also evaluates the behavior through six scales that are based on the diagnostic criteria of DSM-5: affective disorders, anxiety disorders, somatic disorders, attention and hyperactivity disorders, oppositional-provocative disorders and conduct disorders. Behavioral scales are an essential element in the assessment and diagnosis of children with emotional and behavioral problems as they allow for a collection of information from people who have spent months or years with the child.

***SCARED:*** It consists in a self-administered questionnaire consisting of 41 questions that must be answered in the first person, and it assesses the presence of various childhood anxiety disorders (panic disorder, social phobia, generalized anxiety disorder and separation anxiety disorder), as described by DSM-IV. It is designed for children between 8 and 17 years old. SCARED allows the screening of anxiety disorders in research samples, and it is also a useful tool that helps in the diagnosis of anxiety disorders in clinical samples.

***CDI 2:*** It consists in a self-assessment scale of depression that can be administered to subjects from 8 to 17 years of age. The questionnaire evaluates a wide variety of symptoms such as mood disorders, the ability to feel pleasure, vegetative functions, self-esteem and social behavior. Each of the 27 items of the test provides three alternatives answers that the subject is invited to choose based on the ideas and feelings they experienced in the last two weeks. Numerous items specifically investigate the effects of a depressive condition in those contexts that are particularly relevant to the child (e.g., school). The instrument can be useful to define the level of severity of the disease or to determine (even in a school setting) those individuals at risk or who are experiencing a temporary state of depression.

***SES:*** It consists in a self-administered questionnaire that allows to collect information about the level of education and professional level of the parents. It also indicates the position of the person or family within the social system.

### 2.3. Procedures

The subjects included in the sample previously received diagnoses of HFA following a clinical evaluation confirmed with the criteria of DSM-5 [2]. The assessment included: ADOS 2-Module 3 (Autism Diagnostic Observation Schedule), KADI (Krug Asperger’s Disorder Index), K-SADS-PL DSM-5. Clinical evaluations were carried out in January 2020. We then divided the subjects into two groups and performed an initial evaluation of the internalizing symptoms, before carrying out the training (T0), by administering the YSR, SCARED and CDI 2 tests to the boys. The two groups subsequently performed a specific training for 6 months (from February 2020 to July 2020) on a weekly basis, conducted by psychologists specialized in cognitive-behavioral psychotherapy: Gr1 performed a training based on SCT (standard cognitive therapy) while Gr2 performed a training based on mindfulness (M-ERE). Group assignment was randomized.

At the end of the treatment (T1), we re-administered the YSR, SCARED and CDI 2 tests to the participants. We collected the data from the various measurements and investigated any improvements or worsening in order to verify the effectiveness of the two trainings.

***SCT:*** This intervention included a weekly individual meeting. The meetings were initially structured through the collection of ABCs for recording thoughts, and then proceeded towards the cognitive restructuring of beliefs [27].

The protocol provided, in the first two meetings, activities of global psychoeducation to the model, then a phase I intervention dedicated to activities of: recording of thoughts, formulation of ABCs and recognition of dysfunctional automatic thoughts (cognitive distortions). Then phase II, starting from the third month, problem solving activities and identification of coping strategies were carried out for the identification of alternative thoughts to dysfunctional automatic thoughts. From the fourth month until the end of the intervention, restructuring activities were carried out on the intermediate and deep beliefs.

***M-ERE:*** This intervention included a weekly individual meeting. The meetings were initially structured through emotional rational education, which included education in the recognition of emotional expressions, then in the classification and gradation of emotions. Mindfulness was then applied according to the standard Kabat-Zinn protocol [26].

The protocol provided, in the first two meetings, global psychoeducation activities to the model, then an intervention dedicated to activities of: recognition of emotional expressions, labeling of emotions and emotional appropriateness, both in terms of intensity and congruence of the emotional response (emotion thermometer). Starting from the third month, activities were carried out to recognize irrational thoughts, to correlate them with dysfunctional emotions and to use disputing to identify alternative solutions. Starting from the fourth month, for a total of 8 meetings, mindfulness was introduced according to the Kabat-Zinn protocol through activities for raising awareness and strengthening decentralization strategies aimed at improving the quality of disputing. This group followed the activities first of ERE and then of mindfulness by participating in all sessions from the first to the last, for a total of 6 months.

Each session, for both interventions, lasted 60 min. Participants from both groups were present at each session. Participants who would not have been constant were eliminated from the sample before enrollment in the study: participation in all sessions was an initial inclusion criterion (the sample examined in the study is composed of 54 subjects who followed the constant intervention).

During the lockdown, the sessions were held in safety situations, with the permitted distance (one meter) and through the use of masks, as allowed by the Ministerial Decree according to which health services could take place in presence.

The study was conducted according to the guidelines of the Declaration of Helsinki and approved by the Academic Senate and the Ethics Committee of University of International Studies of Rome (UNINT). Written informed consents were obtained from all the parents of the subjects and each participant gave an oral consent to participate in the study.

### 2.4. Data Analysis

Data analyses were performed with SPSS 26.0 [3,40]. Significance was accepted at the 5% level (*p* < 0.05). Dependent sample *t*-test for both groups were performed. Afterwards, a multivariate analysis of covariance was performed with group as the independent variable, post-test scores (T1) as the dependent variables, and pre-test scores (T0) as covariates. Moreover, the three ANCOVAs univariates, one for each measure of interest, were then added.

## 3. Results

**Figure 1:**
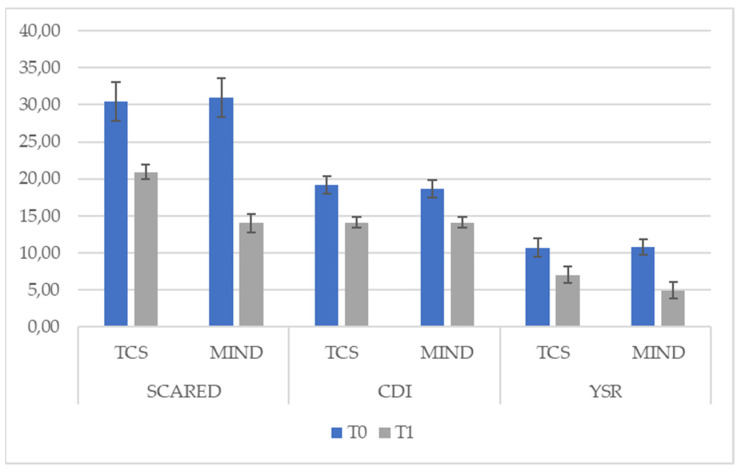
Comparisons between T0 and T1 for the three scales.

Dependent sample *t*-tests for both groups revealed that the Gr2 produced larger t-values than the Gr1 for all three measures and that both groups showed a significant reduction of the scores (see Table 2 and Figure 1).

**Figure 2:**
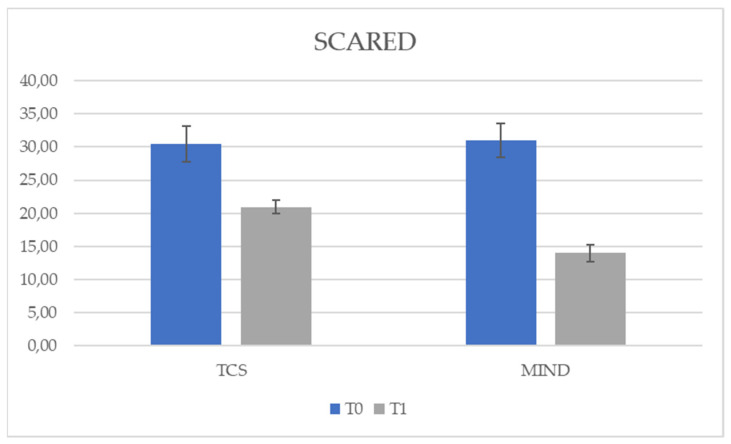
Effect of treatment group in SCARED scale.

**Figure 3:**
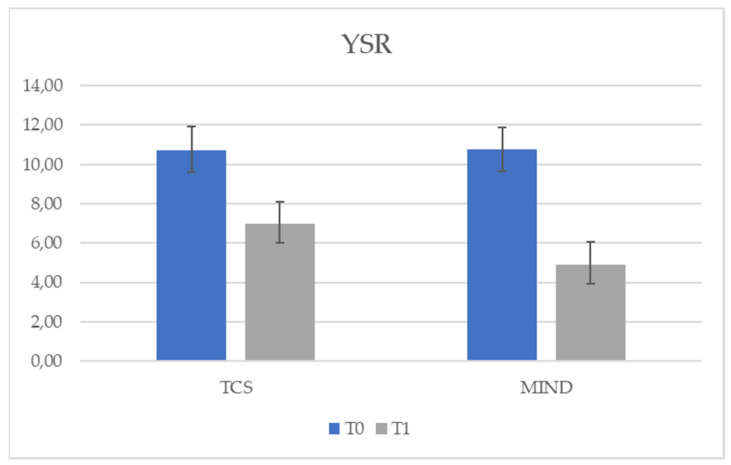
Effect of treatment group in YSR scale.

**Figure 4:**
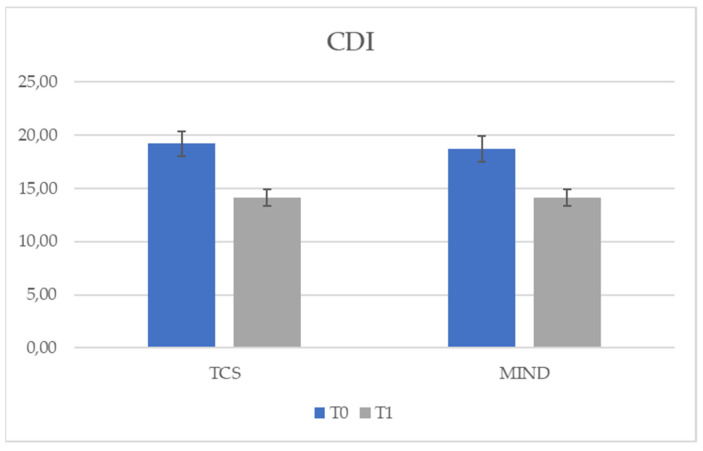
Effect of treatment group in CDI scale.

To further compare the treatments, a multivariate analysis of covariance was performed with group as the independent variable, post-test scores (T1) as the dependent variables, and pre-test scores (T0) as covariates. So, the group factor significantly predicted the post-test scores for SCARED, F (1,49) = 497.69, *p* < 0.05 (see Figure 2), and YSR, F (1,49) = 39.09, *p* < 0.05 (see Figure 3), while the group had no effect on CDI at T1, *p* = 0.69 (see Figure 4). This meant that the two treatments differed in terms of symptoms both for SCARED (Mean TCS = 20.91; Mean MIND =14.02) and YSR scale (Mean TCS = 6.96; Mean MIND = 4.96), so that treatment with mindfulness led to less symptoms.

As regards the three covariates:

− No significant effect of SCARED at T0 was found (*p* > 0.05) on the three post-test scores;
− CDI did not have effect on SCARED and YSR at T1 (*p* > 0.05), but reached significance on CDI at T1, F (1,49) = 4.09, *p* = 0.049;
− YSR did have a significant impact on SCARED at T1, F (1,49) = 4.83, *p* < 0.05, but not on the others (*p* > 0.05).

The individual ANCOVAs conducted later further confirmed these results: only the treatment factor explained the effect on the T1 scores for SCARED, F (1,51) = 485.3, *p* < 0.05, and YSR, F (1,51) = 45.02, *p* < 0.05, but not for the CDI (*p* > 0.05). It seems that the decrease in CDI scores in the post-test was due to the presence of treatment regardless of the type. The covariate effect of CDI at pre-test was significant in the MANCOVA but not in the individual ANCOVA (*p* > 0.05), probably because of the redistribution of explained variance. The effect of the pre-test covariate was not significant (*p* > 0.05) in the other two scales.

To sum up, the *t*-test showed a significant reduction in symptoms with both kinds of treatment (see Table 1) for all symptoms scales (CDI, YSR and SCARED). However, the treatment of Gr2 clearly reduced scores on the YSR and the SCARED questionnaires rather than the treatment of Gr1.

## 4. Discussion

The results of our study using *t*-tests showed a significant reduction in symptoms with both types of treatment (see Table 1) for all symptom scales (CDI, YSR and SCARED). However, by using a multivariate analysis of covariance with the group as an independent variable, the post-test (T1) scores as dependent variables and the pretest (T0) scores as covariates, the group factor significantly predicted scores at T1 for SCARED (see Figure 2) and YSR (see Figure 3), while the group had no effect on CDI at T1 (see Figure 4). Furthermore, the use of the individual ANCOVAs conducted subsequently further confirmed these results: only the treatment factor explained the effect on T1 scores for SCARED and YSR, but not for CDI (*p* > 0.05). It appears that the decrease in CDI scores at T1 was due to the presence of treatment regardless of type. However, M-ERE treatment clearly reduced scores on YSR and SCARED questionnaires rather than SCT treatment. Therefore, our assumptions were supported by the results. Referring to previous studies, there is a lot of research supporting the efficacy of mindfulness-based treatment in reducing anxiety in adults in clinical and non-clinical populations [41,42,43]. However, there are few works that demonstrate the effectiveness of mindfulness in the treatment of internalizing symptoms (anxiety, stress and worries) in adolescents or in subjects with HFA, and there are no studies investigating the effect of the combined treatment of mindfulness and rational emotional education. Children and adolescents affected by HFA appear very vulnerable to social, emotional and behavioral problems; therefore, it is necessary to intervene in order to reduce the levels of stress and anxiety in this population and, also in this case, mindfulness could prove to be an effective treatment. However, data on the efficacy of evidence-based interventions to reduce anxiety in such individuals are limited, especially for adolescents [44]. A study by Keng et al. [43] examined the effectiveness of using mindfulness by applying it to clinical and non-clinical populations, and highlighted reduced levels of anxiety, depression, anger, rumination, general psychological distress, cognitive disorganization and post-traumatic avoidance symptoms. Authors also revealed an improvement in terms of an increased sense of awareness and empathy, satisfaction with life and an improvement in the quality of life of these individuals. Furthermore, such intervention proved to be valid and efficient also in terms of costs and time, as well as helpful both for the subjects and their families. A study conducted with parents of children with HFA investigated the impact of mindfulness-based training [45]; the results showed that parental-reported stress levels dropped significantly for five out of six mothers. Improvements in the quality of family life after treatment have also been reported. Greater parental awareness remained stable in both phases. At the end of parental training, a significant reduction was found in the children’s total problem score on the Child Behavior Checklist (CBCL), along with reductions in the children’s anxious symptomatology and thinking problems.

After parent-mediated child awareness training, further reductions in anxiety and thinking problems was noted. Another study was conducted in parallel with adolescents with HFA and their parents [46] following mindfulness-based training. After the awareness intervention, parents reported an increase in conscious behavior (defined as observing, describing, acting with awareness and non-reactivity), an increase in quality of life and a reduction in parenting stress. Adolescents reported a significant decrease in post-test rumination, which remained at follow-up. Parents reported that their children’s social cognition, social communication and level of worries improved significantly post-test, whereas their social responsiveness improved at follow-up. Despite these promising results, few studies have investigated the effectiveness of implementing mindfulness-based awareness interventions in individuals with HFA, and none are associated with rational emotional education treatment.

Therefore, compared to existing studies, our study contributes to increasing knowledge on the effectiveness of mindfulness-based interventions in these subjects. In fact, in our study there was a general improvement in the internalizing symptoms of the boys both after the SCT treatment and after the M-ERE intervention: more specifically, the treatment with M-ERE clearly reduced the scores on the YSR and SCARED questionnaires relating to the symptoms internalizing/anxious. While, with regard to depressive symptoms (investigated by the CDI), it seems that the decrease in CDI scores at T1 was due to the presence of the treatment regardless of the type. In fact, in our work, while no significant differences are observed between Gr1 and Gr2 as regards CDI at T1, the differences between the two groups regarding SCARED and YSR at T1 appear to be significant. M-ERE treatment for depressive symptoms worked, but the expected results were on par with SCT treatment, which proved to be just as effective.

## 5. Limits and Conclusions

Overall, the results of our work highlight the efficacy of mindfulness-based interventions in the treatment of internalizing symptoms in adolescents with HFA and specifically show that an M-ERE intervention appears to be effective in managing anxiety to a greater extent than treatment with SCT. However, M-ERE intervention appears to be equally effective as SCT treatment in the management of depressive symptoms. Although the outcomes offer insights on the improvement of treatments for HFA, our data need to be considered with caution. In fact, a limitation of our study relates to the possible presence of a test–re-test sensitization and, although six months had passed between one assessment and another, we do not know whether a learning effect occurred and, eventually, in which measure. Moreover, another limitation might refer to the types of psychotherapies compared, being the mindfulness intervention more body-oriented than the SCT. Therefore, future studies might limit the test-retest sensitization and implement treatments that appear comparable to a higher degree. In addition, in order to demonstrate the effectiveness of the treatment, the duration of the follow-up could be increased up to one year with the purpose to verify the maintenance of the skills developed and the possible generalization of the results achieved.

In conclusion, we hope that future studies may aim at evaluating other interventions for internalizing symptoms alleviation; for example, by including more holistic approaches related to the body and not only to the cognitive functions with the overarching aim of improving both the relationships and the quality of life of HFA adolescents.

## Figures and Tables

**Table 1:** Characteristics of the sample.

		**Group 1**			
***ADOS 2***		***WISC IV***		***AGE***	
*Means*	*SD*	*Means*	*SD*	*Means*	*SD*
12	0.53	109.5	0.40	13.50	0.70
		**Group 2**			
***ADOS 2***		***WISC IV***		***AGE***	
*Means*	*SD*	*Means*	*SD*	*Means*	*SD*
12.3	0.45	108.9	0.43	13.30	0.55

**Table 2:** Dependent sample *t*-test for the three questionnaires.

		T0		T1				
Scale	Group	Mean	SD	Mean	SD	t	df	*p*
**SCARED**	**TCS**	30.44	2.665	20.93	0.997	17.130	26	<0.05 *
	**MIND**	30.96	2.564	14.00	1.271	28.716	26	<0.05 *
**CDI**	**TCS**	19.19	1.145	14.11	0.751	19.606	26	<0.05 *
	**MIND**	18.70	1.171	14.11	0.751	21.852	26	<0.05 *
**YSR**	**TCS**	10.70	1.203	7.00	1.109	12.302	26	<0.05 *
	**MIND**	10.78	1.086	4.93	1.141	18.885	26	<0.05 *

* The accepted significance is <0.05. Note: T0 (measurement collected before psychotherapy training), T1 (measurement carried out 6 months after performing psychotherapy training).

## Data Availability

The data is available from the corresponding author.

## References

[1] American Psychiatric Association (2000). DSM-IV-TR: Diagnostic and Statistical Manual of Mental Disorders, Text Revision.

[2] American Psychiatric Association (2013). Diagnostic and Statistical Manual of Mental Disorders.

[3] Frolli A., Bosco A., Di Carmine F., Cavallaro A., Lombardi A., Sergi L., Ricci M.C. (2021). Parent Training and Therapy in Children with Autism. Pediatric Rep..

[4] Farrugia S., Hudson J. (2006). Anxiety in adolescents with Asperger syndrome: Negative thoughts, behavioral problems, and life interference. Focus Autism Other Dev. Disabil..

[5] Kuusikko S., Pollock-Wurman R., Jussila K., Carter A.S., Mattila M.L., Ebeling H., Moilanen I. (2008). Social anxiety in high-functioning children and adolescents with autism and Asperger syndrome. J. Autism Dev. Disord..

[6] Mattila M.L., Hurtig T., Haapsamo H., Jussila K., Kuusikko-Gauffin S., Kielinen M., Moilanen I. (2010). Comorbid psychiatric disorders associated with Asperger syndrome/high-functioning autism: A community-and clinic-based study. J. Autism Dev. Disord..

[7] Lopata C., Toomey J.A., Fox J.D., Volker M.A., Chow S.Y., Thomeer M.L., Smerbeck A.M. (2010). Anxiety and depression in children with HFASDs: Symptom levels and source differences. J. Abnorm. Child Psychol..

[8] Kose L.K., Fox L., Storch E.A. (2018). Effectiveness of cognitive behavioral therapy for individuals with autism spectrum disorders and comorbid obsessive-compulsive disorder: A review of the research. J. Dev. Phys. Disabil..

[9] White S.W., Ollendick T., Albano A.M., Oswald D., Johnson C., Southam-Gerow M.A., Scahill L. (2013). Randomized controlled trial: Multimodal anxiety and social skill intervention for adolescents with autism spectrum disorder. J. Autism Dev. Disord..

[10] Mayes S.D., Calhoun S.L., Murray M.J., Ahuja M., Smith L.A. (2011). Anxiety, depression, and irritability in children with autism relative to other neuropsychiatric disorders and typical development. Res. Autism Spectr. Disord..

[11] Bachevalier J., Loveland K.A. (2006). The orbitofrontal–amygdala circuit and self-regulation of social–emotional behavior in autism. Neurosci. Biobehav. Rev..

[12] Reichow B., Volkmar F.R. (2006). Social skills interventions for individuals with autism: Evaluation for evidence-based practices within a best evidence synthesis framework. J. Autism Dev. Disord..

[13] Solomon M., Goodlin-Jones B.L., Anders T.F. (2004). A social adjustment enhancement intervention for high functioning autism, Asperger’s syndrome, and pervasive developmental disorder NOS. J. Autism Dev. Disord..

[14] Mazzone L., Postorino V., De Peppo L., Fatta L., Lucarelli V., Reale L., Vicari S. (2013). Mood symptoms in children and adolescents with autism spectrum disorders. Res. Dev. Disabil..

[15] Hoffmann W., Weber L., König U., Becker K., Kamp-Becker I. (2016). The role of the CBCL in the assessment of autism spectrum disorders: An evaluation of symptom profiles and screening characteristics. Res. Autism Spectr. Disord..

[16] Burkhart K., Knox M., Hunter K. (2018). Cognitive-behavioral therapy in the treatment of internalizing disorders in high-functioning youth with autism spectrum disorder. J. Contemp. Psychother..

[17] Keefer A., White S.W., Vasa R.A., Reaven J. (2018). Psychosocial interventions for internalizing disorders in youth and adults with ASD. Int. Rev. Psychiatry.

[18] Sofronoff K., Attwood T., Hinton S. (2005). A randomised controlled trial of a CBT intervention for anxiety in children with Asperger syndrome. J. Child Psychol. Psychiatry.

[19] Wood J.J., Drahota A., Sze K., Van Dyke M., Decker K., Fujii C., Spiker M. (2009). Brief report: Effects of cognitive behavioral therapy on parent-reported autism symptoms in school-age children with high-functioning autism. J. Autism Dev. Disord..

[20] Cardaciotto L., Herbert J.D. (2004). Cognitive behavior therapy for social anxiety disorder in the context of Asperger’s syndrome: A single-subject report. Cogn. Behav. Pract..

[21] Lang R., Regester A., Lauderdale S., Ashbaugh K., Haring A. (2010). Treatment of anxiety in autism spectrum disorders using cognitive behaviour therapy: A systematic review. Dev. Neurorehabilit..

[22] Frolli A., La Penna I., Cavallaro A., Ricci M.C. (2019). Theory of Mind: Autism and Typical Development, Acad. J. Ped. Neonatol..

[23] Ellis A. (2006). Rational emotive behavior therapy and the mindfulness based stress reduction training of Jon Kabat-Zinn. J. Ration. Emot. Cogn. Behav. Ther..

[24] Kabat-Zinn J. (2015). Mindfulness. Mindfulness.

[25] Sizoo B.B., Kuiper E. (2017). Cognitive behavioural therapy and mindfulness based stress reduction may be equally effective in reducing anxiety and depression in adults with autism spectrum disorders. Res. Dev. Disabil..

[26] Ridderinkhof A., de Bruin E.I., Blom R., Bögels S.M. (2018). Mindfulness-based program for children with autism spectrum disorder and their parents: Direct and long-term improvements. Mindfulness.

[27] Clark D.A., Beck A.T. (2011). Cognitive Therapy of Anxiety Disorders: Science and Practice.

[28] Di Pietro M. (1999). L’ABC delle mie emozioni. Corso di alfabetizzazione socio-affettiva. Manuale per L’Alunno.

[29] Di Pietro M. (2014). ABC delle Mie Emozioni.

[30] Ellis A. (1995). Changing rational-emotive therapy (RET) to rational emotive behavior therapy (REBT). J. Ration. Emot. Cogn. Behav. Ther..

[31] David D. (2014). Rational emotive behavior therapy (REBT). The Encyclopedia of Clinical Psychology.

[32] Orsini A., Pezzuti L., Picone L., Giunti O.S. (2012). WISC-IV: Contributo Alla Taratura Italiana (WISC-IV Italian).

[33] Achenbach T.M., Rescorla L.A. (2001). Manual for the ASEBA School-Age Forms and Profiles.

[34] Kovacs M. (2011). Children’s Depression Inventory 2nd Edition (CDI 2): Technical Manual.

[35] Crocetti E., Hale W.W., Fermani A., Raaijmakers Q., Meeus W. (2009). Psychometric properties of the Screen for Child Anxiety Related Emotional Disorders (SCARED) in the general Italian adolescent population: A validation and a comparison between Italy and The Netherlands. J. Anxiety Disord..

[36] Venuti P., Senese V.P. (2007). Un questionario di autovalutazione degli stili parentali: Uno studio su un campione italiano. G. Di Psicol..

[37] Lord C., Rutter M., Di Lavore P.C., Risi S., Gotham K., Bishop S. (2012). Autism Diagnostic Observation Schedule: ADOS-2.

[38] Krug D.A., e Arick J.R. (2007). KADI/Krug Asperger’s Disorder Index.

[39] Kaufman J., Birmaher B., Axelson D., Perepletchikova F., Brent D., e Ryan N. (2016). “K-SADS-PL DSM-5.” Child and Adolescent Research and Education.

[40] Corp, IBM (2019). Statistiche IBM SPSS Per Windows.

[41] Biegel G.M., Brown K.W., Shapiro S.L., Schubert C.M. (2009). Mindfulness-based stress reduction for the treatment of adolescent psychiatric outpatients: A randomized clinical trial. J. Consult. Clin. Psychol..

[42] Burke C.A. (2010). Mindfulness-based approaches with children and adolescents: A preliminary review of current research in an emergent field. J. Child Fam. Stud..

[43] Keng S.L., Smoski M.J., Robins C.J. (2011). Effects of mindfulness on psychological health: A review of empirical studies. Clin. Psychol. Rev..

[44] Schall C.M., McDonough J.T. (2010). Autism spectrum disorders in adolescence and early adulthood: Characteristics and issues. J. Vocat. Rehabil..

[45] Hwang Y.S., Kearney P., Klieve H., Lang W., Roberts J. (2015). Cultivating mind: Mindfulness interventions for children with autism spectrum disorder and problem behaviors, and their mothers. J. Child Fam. Stud..

[46] De Bruin E.I., Blom R., Smit F.M., van Steensel F.J., Bögels S.M. (2015). MYmind: Mindfulness training for youngsters with autism spectrum disorders and their parents. Autism.

